# What Happens in the Staphylococcal Nucleoid under Oxidative Stress?

**DOI:** 10.3390/microorganisms7120631

**Published:** 2019-11-29

**Authors:** Kazuya Morikawa, Yuri Ushijima, Ryosuke L. Ohniwa, Masatoshi Miyakoshi, Kunio Takeyasu

**Affiliations:** 1 Faculty of Medicine, University of Tsukuba, Tsukuba, Ibaraki 305-8575, Japan; 2 Department of Emerging Infectious Diseases, Institute of Tropical Medicine (NEKKEN), Nagasaki University, Nagasaki 852-8523, Japan; 3 Graduate School of Biostudies, Kyoto University, Yoshida-Konoe, Sakyo-ku, Kyoto 606-8501, Japan

**Keywords:** *Staphylococcus aureus*, *Escherichia coli*, oxidative stress, nucleoid, MrgA, Dps, nucleoid associated protein, atomic force microscopy

## Abstract

The evolutionary success of *Staphylococcus aureus* as an opportunistic human pathogen is largely attributed to its prominent abilities to cope with a variety of stresses and host bactericidal factors. Reactive oxygen species are important weapons in the host arsenal that inactivate phagocytosed pathogens, but *S. aureus* can survive in phagosomes and escape from phagocytic cells to establish infections. Molecular genetic analyses combined with atomic force microscopy have revealed that the MrgA protein (part of the Dps family of proteins) is induced specifically in response to oxidative stress and converts the nucleoid from the fibrous to the clogged state. This review collates a series of evidences on the staphylococcal nucleoid dynamics under oxidative stress, which is functionally and physically distinct from compacted *Escherichia coli* nucleoid under stationary phase. In addition, potential new roles of nucleoid clogging in the staphylococcal life cycle will be proposed.

## 1. Introduction

The application of atomic force microscopy (AFM), providing direct observation of bacterial nucleoids, has given informative clues that are followed by critical findings in the molecular mechanisms and physiology of prokaryotic systems [1,2]. Nucleoids that are experimentally dispersed from lysed cells are usually observed as fibrous structures in both Gram-positive and Gram-negative bacteria, and also in organelles of prokaryotic origins [3,4]. However, the components of the nucleoids are diverse among bacterial species and their amounts and constituent components undergo dynamic changes depending upon environmental conditions. Such dynamic behavior of the nucleoid components can be linked to the transition of physical characteristics of the nucleoid. This short review summarizes what is known about the staphylococcal nucleoid, especially focusing on its unique morphological change under oxidative stress, and discusses its potential relevance in the life of this important human pathogen.

## 2. *Staphylococcus aureus* Lifestyle and Importance of Oxidative Stress Resistance

*Staphylococcus* belongs to the Gram-positive *Bacilli* class of Firmicutes that contains a low G/C content in the genome. This class also includes *Bacillus* and *Listeria* spp. The genus *Staphylococcus* is composed of about 60 species [5], and the most clinically relevant one is *S. aureus. S. aureus* asymptomatically inhabits our nasal cavity but is a major opportunistic pathogen responsible for a broad spectrum of infections ranging from superficial skin abscesses to more severe life-threatening diseases such as pneumonia, sepsis, and toxic shock syndrome. Staphylococcal infections, both in hospitals and in the community, are serious problems in clinical settings, largely because of the difficulty in antibiotic treatment arising due to acquired resistance [6].

*S. aureus* has to cope with a variety of environmental stresses and bactericidal factors in host environments [7]. These include desiccation, hyperosmolarity [8,9,10], and the immune system [11,12]. Once *S. aureus* is phagocytosed, it is challenged by a series of host bactericidal factors such as acidic pH, antimicrobial peptides, and reactive oxygen species (ROSs). A series of host enzymes and “Fenton reaction” are responsible for the generation of ROSs. NADH oxidase generates superoxide anion from oxygen [13] while superoxide dismutase (SOD) catalyzes its conversion into hydrogen peroxide [14,15]. Ferrous iron (Fe^2+^) then converts the hydrogen peroxide into the highly reactive hydroxyl radical (this process is called the Fenton reaction) [16,17].

*S. aureus* can survive in professional phagocytes such as neutrophils and macrophages [18,19,20], where the staphylococcal antioxidant enzymes responsible for the detoxification of ROSs must play critical roles. The anti-oxidant enzymes include SOD [21,22], catalase (that converts hydrogen peroxide into H_2_O and O_2_ [23]), and the metallo regulon gene A (MrgA) [24]. It is considered that the ability to survive in migratory phagocytes allows *S. aureus* to spread within our body and to induce severe recurrence or chronic infection [19]. This process relies on multiple regulatory factors, such as Agr (quorum sensing accessory gene regulator) and SigB (general stress response sigma factor), but not SarA (global regulator) [19]. Sortase A and virulence factors such as alpha-toxin, aureolysin, protein A, are also involved in this process [19]. Thus, fine-tuning of the relevant gene expression is necessary in the initial and the following phases of the infection.

## 3. Oxidative Stress Induces Nucleoid Clogging

### 3.1. Characteristics of S. aureus Nucleoid in Comparison with Other Bacteria

Most bacterial genomes are circular. In bacteria, genomic DNA (in the scale of a few cm) is packed in a cell (with a diameter of a few µm) in the form of the “nucleoid” with a variety of proteins, RNAs, low-molecular weight compounds, etc. [25]. In contrast to the interphase eukaryotic genome that is separated by the nuclear envelope from the cytosol, the prokaryotic genome is established in the cytosol without a nuclear envelope; i.e., the prokaryotic genome function is achieved in harmony with replication, transcription, and translation all occurring in the cytosolic environment. A variety of methods to isolate the nucleoid have demonstrated different aspects of nucleoid structures, nucleoid-associated proteins (NAPs), role of RNA, and low molecular weight compounds, etc. For example, electron microscopy observations of the nucleoid isolated under high salt conditions have revealed that the circular fibrous genome in bacteria, as a whole, is bundled in the core portion and forms a rosette-like structure with interwound loops emanating radially from the core [26,27,28].

**Figure 1:**
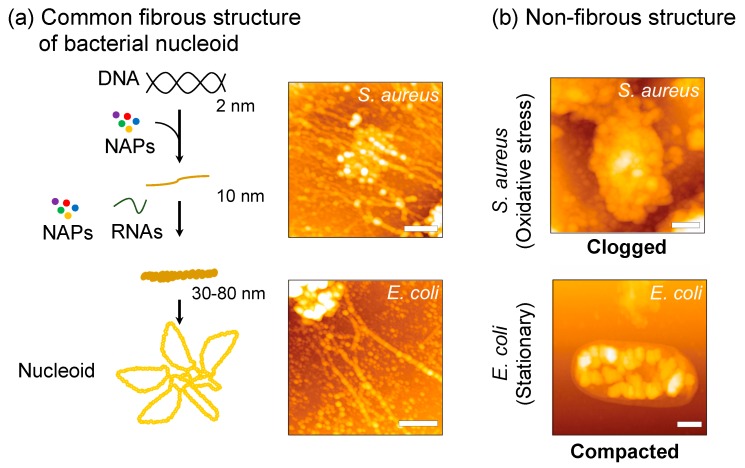
Nucleoid structure and dynamics in bacteria. (**a**) A model of the structural hierarchy of bacterial nucleoid proposed by a series of dissection analyses with AFM and on-substrate lysis method [2]. Naked DNA (2 nm thickness) is complexed with nucleoid-associated proteins (NAPs) to form fibers 10 nm in width, which are a fundamental structural unit to form thicker fibers as well as the compacted nucleoid [31]. Scale bar: 500 nm. (**b**) Non-fibrous structures. Staphylococcal nucleoid is clogged under oxidative stress, but not in the stationary phase. In contrast, *E. coli* nucleoid is compacted in the stationary phase. Scale bar: 500 nm. Original source of AFM images is [7].

The nucleoid released from cells lysed under physiological salt concentrations is observed as a fibrous structure with variable thickness regardless of the bacterial species: *S. aureus*, *Escherichia coli*, and *Clostridium perfringens* [3] (Figure 1a). The fiber thickness ranges between 30~80 nm in width with NAPs and RNAs as structural components [29]. Treatment of the released nucleoids by RNase A, which digests mainly single-stranded RNA [30], makes the nucleoid fibers narrow down to 10 nm, but never releases the naked DNA (2 nm) [29]. In addition, neither RNase III nor RNase H can release the 10-nm fibers. Treatment with rifampicin that targets RNA polymerase to prohibit the transcription also increases the proportion of 10-nm fibers. Thus, nascent RNAs and single-stranded RNAs are involved in the 30~80 nm fibrous nucleoid. It is likely that RNAs are interwoven to thicker fibers in the released nucleoid structure. These hierarchical organizations seem to be general characteristics of bacterial nucleoid. RNase treatment also converts thick nucleoid fibers to thinner ones in the organelles of prokaryotic origin; i.e., chloroplasts and mitochondria [4].

Protease treatment of *E. coli* nucleoid releases not only 10-nm fibers but also naked DNA [29], suggesting that NAPs are structurally important components in nucleoid organization. The *E. coli* nucleoids isolated under mild salt concentrations with spermidine consist of a set of DNA-binding proteins including the RNA polymerase subunits and about 300 species of transcription factors [32,33]. Among them, Hu (heat-unstable nucleoid protein), HNS (histone-like nucleoid structuring protein), IHF (integration host factor protein), StpA (suppressor of T4 *td* mutant phenotype A, H-NS homolog), Dps (DNA-binding protein from starved cells), Fis (factor for inversion stimulation), and Hfq (host factor for phage RNA Qβ replication) were historically believed to be the major nucleoid proteins that were structurally and functionally important [34]. Hfq is now recognized as an RNA chaperone that governs post-transcriptional regulation [35], although another role of Hfq has been implicated in plasmid replication, transposition, and transcription [36]. On the other hand, Hfq was shown to alter the DNA topology indirectly rather than directly associating with DNA [37]. Here it is interesting to note that the released nucleoid from lysed cells of single deletion mutant strains of *E. coli* (i.e., deletion mutants of genes encoding Hu (*hupA*, *hupB*), HNS (*hns*), IHF (*himA*, *himD*), StpA (*stpA*), Fis (*fis*), and Hfq (*hfq*)) sustained the fiber structure of 10 nm [29]. This result suggests that each protein is not essential by itself to build up 10-nm fibers.

These proteins are shared among Gram-negative bacteria. However, other than Hu and Dps homologues, they are missing in the genomes of Gram-positive bacteria including *S. aureus* [2]. Namely, irrespective of the structural similarity of the nucleoids mentioned above, many NAPs are diverse depending on the species [38]. *S. aureus* has an Hfq homologue with a substantial RNA binding activity [39]. However, its function still remains elusive since its deletion exhibits no phenotypic changes [40].

Subtractive proteomic analysis of the nucleoid isolated under physiological salt concentrations with spermidine identified staphylococcal proteins that exclusively exist in the nucleoid fraction, but not in soluble cytosol and membrane fractions. They were termed csNAPs (contamination subtracted list of NAPs). The complete lists of 92 csNAPs-log (log phase), 141 csNAPs-st (stationary phase), and 113 csNAPs-ox (oxidative stress) are available in [38,41]. The top 50 csNAPs, sorted by the emPAI values that reflect the protein abundance, are summarized in Table 1. Staphylococcal csNAPs contains global regulators, fatty acid synthesis enzymes, oxidoreductases, and ribosomal proteins [41], which are common features in bacterial nucleoids [38], and is reasonable if we consider the environmental differences between prokaryotic and eukaryotic genomes (i.e., the absence and presence of nuclear envelope).

### 3.2. Apparent Correlation between Nucleoid Clogging and Oxidative Stress

**Figure 2:**
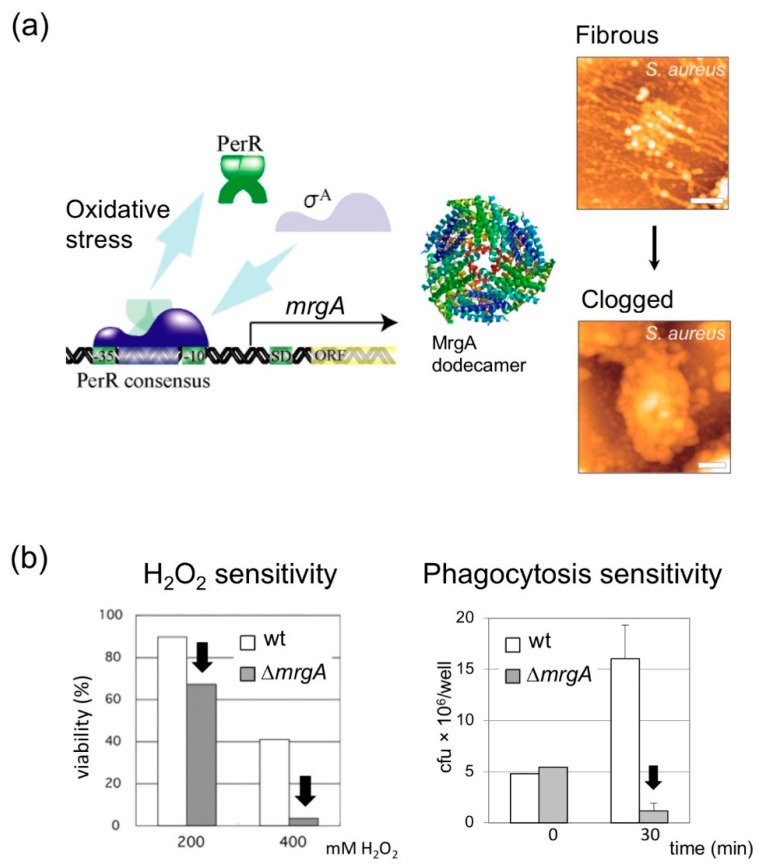
MrgA is essential for the oxidative stress response. (**a**) Left: A model of *mrgA* gene regulation. The *mrgA* gene is among the highly up-regulated genes upon phagocytosis [18]. PerR, the suppressor of *mrgA* transcription, can sense oxidative stress and dissociate from the *mrgA* promoter to release the inhibition. By this regulation, MrgA is specifically expressed under oxidative stress conditions and induces the nucleoid clogging [42]. Center: MrgA forms dodecamer like other Dps family proteins [24]. It lacks known DNA binding regions, and how MrgA binds DNA is not known [43]. Right: AFM images of nucleoid dynamics. Scale bar: 500 nm. (**b**) The *mrgA* gene is essential in hydrogen peroxide resistance [42], as well as in phagocytosis resistance [24]. These resistances are attributed to the ferroxidase activity of MrgA [24]. A *mrgA* deletion increased the sensitivities to H_2_O_2_ (left) and the time-dependent phagocytic killing (right). Error bars at the 30 min time point represent SD (*n = 3*). Images and graph data were reproduced from [7,24,42].

In *S. aureus*, the fibrous structures released from lysed cells diminish under oxidative stress conditions and the nucleoids are observed as clogged forms [42] (Figure 1b). The key factor to cause such clogging was found to be MrgA (similar to Dps family proteins in *E. coli*, see Section 3). The *mrgA* gene does not express its gene product without oxidative stress due to transcription suppression by PerR (Figure 2a). Once PerR senses the oxidative stress, it is released from the *mrgA* promoter and *mrgA* transcription is induced. Owing to this tight regulation, MrgA is specifically expressed under oxidative stress conditions, and reaches c.a. 30,000 molecules (2500 dodecamer) per cell [43]. The deletion mutant of *mrgA* is unable to clog the nucleoid under oxidative stress, while artificial over-expression of MrgA by plasmid, or by mutation in the *perR* suppressor gene, results in the nucleoid clogging even under normal growth conditions without the oxidative stress.

Similar, but physiologically and physically distinct, changes in nucleoid dynamics have been observed in *E. coli* (reviewed in [2]) (Figure 1b), where Dps plays a key role. The expression of Dps in *E. coli* is induced by oxidative stress (as a part of the OxyR regulon) as well as in the stationary phase. Dps is the dominant nucleoid protein in the stationary phase [44], and the nucleoid is tightly compacted [45], limiting the access of DNA binding proteins (except for RNA polymerase [46]). However, Dps expression in the log-phase does not compact the nucleoid because a log-phase dominant nucleoid protein, Fis, prevents the compaction [47]. In contrast to *E. coli*, artificial expression of MrgA by plasmid in *S. aureus* results in clogged nucleoid irrespective of the growth phases. The MrgA-expressing cells are not different in the growth rate from the wild type cells, indicating that nucleoid clogging does not prohibit genome functions such as replication and gene expression. Thus, nucleoid clogging in response to oxidative stress seems to be a phenomenon specific in *S. aureus*, of which physiological relevance is still open to discussion (see the following sections).

## 4. Is Nucleoid Clogging Required or Not for the Oxidative Stress Tolerance?

### 4.1. MrgA Is a Bifucntional Molecule with Ferroxidase Activity That Is Essential for Oxidative Stress Resistance

*S. aureus* MrgA is important for oxidative stress resistance like other Dps family proteins [42] (Figure 2b). Dps family proteins usually assemble into dodecamers and exert ferroxidase activity. MrgA also assembles into dodecamers and the structural data is available in Protein Data Bank under the accession number of 2D5K [24]. Several, but not all, of Dps family proteins including *E.coli* Dps [48] and staphylococcal MrgA [24] can bind DNA. Scavenging free iron is important to prevent the Fenton reaction that generates the hydroxyl radical from ferrous iron (Fe^2+^) and hydrogen peroxide [49]. There is a report showing that the ferroxidase activity alone, without the DNA binding activity, can contribute to oxidative stress resistance: *Streptococcus mutans* Dpr (Dps-like peroxide resistance gene, Dps-family protein) that can bind iron but not DNA is critical to cope with oxidative stress [50,51].

In *S. aureus*, when the ferroxidase center of MrgA (Asp56 and Glu60) is mutated, the susceptibility to oxidative stress increases [24]. These mutations do not disrupt dodecamer formation and DNA binding activity. Therefore, it can be concluded that; (1) ferroxidase activity is essential, and; (2) DNA binding activity alone is not important for oxidative stress resistance.

### 4.2. DNA Binding Activity of MrgA Is Dispensable for Hydrogen Peroxide Resistance and Survival in Phagosome, but Not for Nucleoid Clogging

While it became evident that the ferroxidase activity of MrgA is important for oxidative stress resistance in *S. aureus* [24], the relevance of DNA binding of MrgA has still been under question. The first point we addressed was whether or not, in addition to the ferroxidase activity, DNA binding of MrgA is essential for the physical protection of the genomic DNA [43]. One difficulty is that the DNA binding domain of MrgA has not been identified, whereas that of *E. coli* Dps is known to be in the N-terminal region [43]. Since we have been unable to make specific MrgA variants that lack the DNA binding activity so far, we instead introduced the N-terminal-deletion mutant of *E. coli* Dps (∆18-Dps) that has no DNA binding activity into the *S. aureus mrgA*-knockout mutant. The obtained results clearly demonstrated that the nucleoid is clogged by the expression of Dps in *S. aureus* ∆*mrgA*, but not by ∆18-Dps, indicating that DNA binding activity of Dps is necessary for nucleoid clogging. By analogy, MrgA DNA binding activity is likely responsible for the nucleoid clogging. In addition, ∆18-Dps, as well as Dps, compensated for MrgA in hydrogen peroxide resistance regardless of nucleoid clogging, demonstrating that the DNA binding activity is dispensable for such resistance itself. Namely, the molecular mechanisms of DNA clogging and hydrogen peroxide resistance are likely to be independent, although both mechanisms may cross-over, depending upon the environmental conditions.

Furthermore, an interesting implication is that the apparently distinct nucleoid clogging in *S. aureus* and nucleoid condensation in *E. coli* are brought about by similar molecular mechanisms. In other words, MrgA and Dps can be exchanged in *S. aureus* for nucleoid clogging. This may be a key feature for further investigation of the molecular mechanisms for genome condensation in general.

## 5. Any Physiological Relevance in Nucleoid Clogging?

### 5.1. Characteristics of csNAPs in the Clogged and Relaxed Nucleoid

According to the list of csNAPs in nucleoids (Table 1), some specific features in the clogged nucleoid can be extracted. First, Hu, an *E. coli* major NAP, always exists as csNAPs in staphylococcal nucleoid regardless of the growth phases or the presence of oxidative stress. Second, other *E. coli* major NAPs are lost through the evolutionary processes in *S. aureus*. Third, on the other hand, the isolated staphylococcal nucleoid contains so-called global regulators (Sar homologues and Rot). These would be the evolutionary distinct staphylococcal counterparts of the *E. coli* major NAPs.

These global regulators are constitutively expressed components of the nucleoid in any conditions (log, stationary, and oxidative stress). Such steady state expression of *S. aureus* global regulators makes a striking contrast to the drastic exchange of *E. coli* major NAPs from the log (Fis abundant) to the stationary phase (Dps dominant) [52]. Upon oxidative stress, Sar homologues are maintained in the nucleoid, but some up- and down-regulations among the homologues may take place (see Table 1). The enzymes responsible for detoxification of oxidative stress are also constitutively detected as csNAPs, although the molecular species are diverse depending on the conditions.

As mentioned above, the *S. aureus* nucleoid clogged by MrgA is biologically active and allows cell proliferation. In fact, ribosomal proteins are abundant csNAPs in the clogged nucleoid. In clear contrast, the compacted *E. coli* nucleoid has few ribosomal proteins [38]. The dynamics of csNAPs upon *S. aureus* nucleoid clogging seems to be less drastic than those in *E. coli* nucleoid compaction. Thus, considerable parts of the nucleoid function are sustained in the clogged form.

### 5.2. Effect of Nucleoid Clogging on Transcriptome Profile

**Figure 3:**
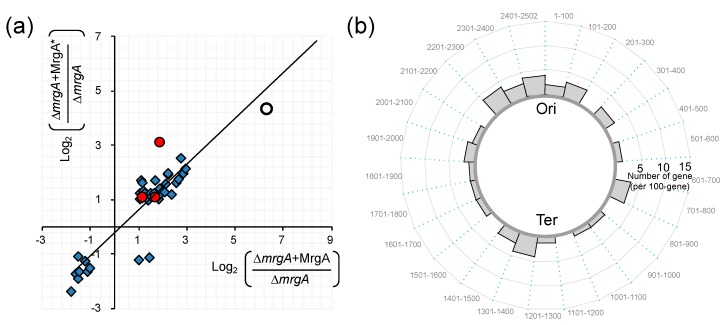
(**a**) Gene expression is affected by MrgA as well as MrgA* similarly in the absence of oxidative stress. Transcriptome data of “*mrgA* deletion mutant (∆*mrgA*)”, “*mrgA* overexpression (∆*mrgA*+MrgA)”, and “mrgA* overexpression (∆*mrgA*+MrgA*)” strains grown in the absence of oxidative stress were obtained by a standard procedure by using GeneChip (Affymetrix). X axis: Comparison between “∆*mrgA*+MrgA” and “∆*mrgA*”. Y axis: Comparison between “∆*mrgA*+MrgA*” and “∆*mrgA*”. Log_2_ fold differences of the loci that showed significant differences (i.e., >2 fold or <0.5 fold) in both comparisons were plotted. Red circles: Protein coding sequences (CDSs). Blue diamonds: Non-CDSs. Open circle: *mrgA*. Thus, MrgA dependent nucleoid clogging can affect the expression of RNAs mainly from non-CDSs in the absence of the oxidative stress. This effect is not due to the ferroxidase activity of MrgA, since the MrgA* overexpression has similar effects to the MrgA overexpression: The correlation coefficient is 0.897. (**b**) Location of the genes which were affected by both MrgA and MrgA* in the absence of oxidative stress. The cumulative numbers of the genes (plotted in graph (**a**)) per 100-gene region are plotted in a circular way. SA numbers in N315 genome are shown outside the circle: 1 = *SA0001* (*dnaA*) through *SA2502*.

Staphylococcal genome is about 2.8 Mbp and contains c.a. 2500 protein-coding genes (c.a. 85% of the genome) [53]. Interestingly, artificial expression of MrgA or MrgA* (MrgA carrying mutations in the ferroxidase centre at Asp56 and Glu60) by plasmid in the ∆*mrgA* strain can affect the transcriptome profile similarly in the absence of oxidative stress (Ushijima et al., in preparation for submission): There were 41 signals significantly changed (>2 fold or <0.5 fold) by MrgA and MrgA*, and MrgA and MrgA* had the same effect for 39 of them (Figure 3). Most of these signals originated from non-coding sequences (Figure 3, diamonds), and only a few from protein coding sequences (Figure 3, red circles). This observation may reflect the differential expression of small RNAs or the difference in the lengths of mRNAs’ untranslated regions. It should be noted that the DNA binding activity of MrgA affected the transcriptome without its ferroxidase activity.

**Figure 4:**
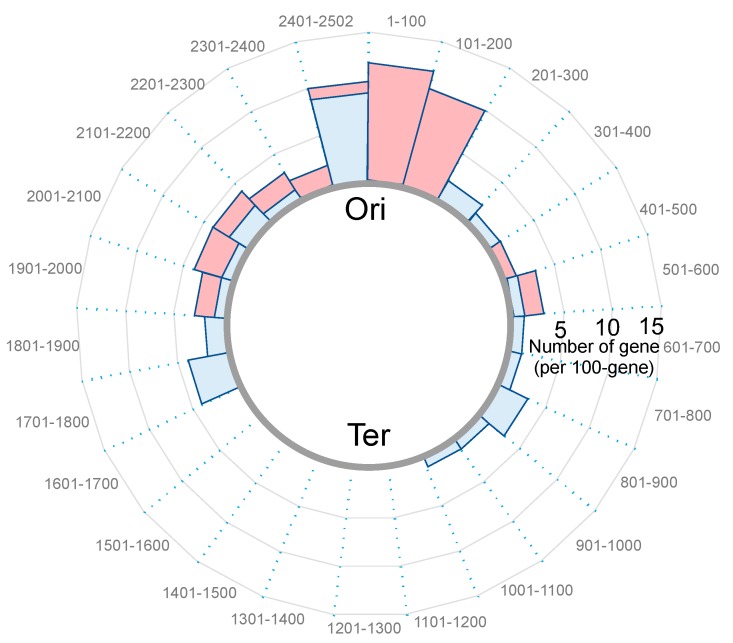
Location of the genes which are differently expressed between WT(+PQ) and ∆*mrgA*(+PQ) under oxidative conditions. The cumulative numbers of the genes (listed in Table 2 and Table 3) per 100-gene region are plotted in a circular way. SA numbers in N315 genome are shown outside the circle: 1 = *SA0001*(*dnaA*), through *SA2502*. Genes that were more (blue; Table 2) or less (red; Table 3) expressed in WT(+PQ) than ∆*mrgA*(+PQ) tend to locate around the Ori-side of the genome.

On the other hand, 112 protein coding genes were up-regulated and 90 were down-regulated under oxidative stress [54]. Under oxidative stress (20 µM PQ: Phenanthrenequinone, [55,56]) WT and ∆*mrgA*, which have clogged and fibrous nucleoids respectively, exhibit distinct profiles in their transcriptomes (91 loci > 2-fold, and 87 loci < 0.5-fold) (Ushijima et al., in preparation for submission). In contrast to Figure 3 (in the absence of oxidative stress), many transcripts from coding sequences were differentially accumulated (listed in Table 2 and Table 3). An intriguing feature of this list is the location dependency; many of the genes are located around the replication origin (Ori), and few from around the Ter side (Figure 4). Notably, the expression patterns of genes in the Staphylococcal Cassette Chromosome (SCC) that locates near the Ori were largely distinct between WT(+PQ) and ∆*mrgA*(+PQ). The lists also present the genes for virulence factors (red), nucleic acid metabolism (green), iron metabolism (*sirC*, *SA0120*), transcription regulators (yellow) including three global regulators (staphylococcal accessory regulators, *sarH1*, *sarY*, *sarV*), and bacteriophage holin/anti-holin.

The results from these analyses would define our next strategies towards understanding what is really going on in the cells before and after oxidative stresses. Considerations on the gene regulatory mechanisms before and after nucleoid clogging under oxidative stress conditions will be accelerated, where a series of oxidative stress responsive regulators (such as PerR, MgrA, SarZ, etc) are cooperatively working [57].

In summary: (1) Under no oxidative stress condition, MrgA binding to nucleoid up-regulates specific non-protein coding genes around the whole genome. (2) Under the stress condition, MrgA binding leads to the up-regulation of the protein coding genes near the Ori. (3) Under the stress condition at the same time, MrgA binging down-regulates the protein coding genes near the Ori. These observations clearly lead us to a few interesting implications. First, MrgA binding may cause different nucleoid status with and without oxidative stress. The evidence for this relies on Figure 3 and Figure 4 as well as Table 2 and Table 3. Second, one structural or physiological conformation is favored for the expression of non-protein coding genes, and the other is preferred by the up- and down-regulations of specific genes. However, the subtleties of certain distinct gene regulations are not known and left as future questions. Third, most nucleoid functions are supposed to be sustained before and after the nucleoid clogging: In this sense, it is interesting to note that we previously described the ‘Armor hypothesis’ by postulating the importance of the localization of antioxidant factors in the nucleoid for genome DNA protection [38].

In conclusion, staphylococcal nucleoid is distinct from the well-studied *E. coli* nucleoid in its dynamics of NAP composition and morphologies. Staphylococcal MrgA is specifically expressed under oxidative stress conditions where it plays important roles to support the survival of this opportunistic human pathogen. So far, any role of nucleoid clogging has not been postulated in the oxidative stress resistance. However, it is now clear that the nuclear clogging represents at least two different structural and functional states of the genome; i.e., under physiological oxidative stress and under the experimental absence of oxidative stress (although whether nucleoid clogging exists physiologically without oxidative stress is still unknown).

**Figure 5:**
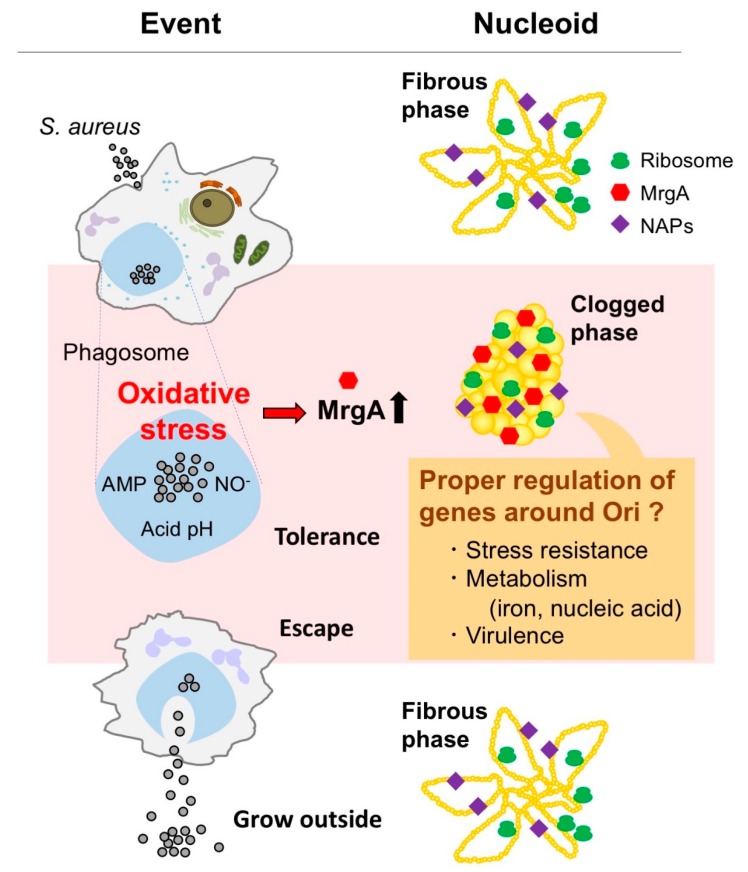
Summary and current hypothesis. *S. aureus* is challenged by oxidative stress in phagosomes. The oxidative stress signal is sensed by the PerR transcriptional repressor leading to the induction of MrgA which converts the nucleoid to the clogged state. Unlike condensed *E. coli* nucleoid, the clogged nucleoid sustains the activities of replication and gene expression that are necessary for cell proliferation. Indeed, the clogged nucleoid retains ribosomes and NAPs including the Sar/Rot global regulators. Nucleoid clogging plays no known role in oxidative stress resistance, but it may be that the clogging phase is preferable for the proper expression of the genes locating around the Ori in the genome. Such gene expression is expected to be involved in the tolerance to phagosome-associated stresses, virulence, and prophage activation. It may also affect other nucleoid-related functions.

A current hypothetical scenario illustrates a certain nucleoid status where gene expression is controlled through the pathogenesis of *S. aureus* (Figure 5). Upon phagocytosis, *S. aureus* senses oxidative stress and induces the expression of MrgA. While the ferroxidase activity directly contributes to the oxidative stress resistance, the DNA binding activity of MrgA converts the nucleoid status into the clogged phase. This may be a preferable state for the proper control of gene expression for survival in phagosomes, as well as preparation for the next step of pathogenesis. Also, it will be an exciting challenge to clarify how particular nucleoid-clogging state is linked to specific gene regulation at the molecular level.

## Figures and Tables

**Table 1:** 50 csNAPs sorted by emPAI values in each condition.

**Oxidative Stress**			
**ID**	**Gene**	**Annotation**	**emPAI**
sau:SA1414	*rpsT*	30S ribosomal protein S20	3.45
sau:SAS033	*rpmF*	50S ribosomal protein L32	3.34
sau:SA0092		hypothetical protein	3.23
sau:SA2032	*rplR*	50S ribosomal protein L18	2.72
sau:SA1663		UPF0342 protein SA1663	1.82
sau:SA0093		hypothetical protein	1.55
sau:SA1504	*infC*	Translation initiation factor IF-3	1
sau:SA2043	*rpsS*	30S ribosomal protein S19	0.89
sau:SA1074	*fabG*	3-oxoacyl-[acyl-carrier-protein] reductase	0.72
sau:SA1279	*gpsB*	Cell cycle protein gpsB	0.69
sau:SA2022	*rplQ*	50S ribosomal protein L17	0.65
sau:SA2062	*sarV*	HTH-type transcriptional regulator sarV	0.64
sau:SA1404	*rpsU*	30S ribosomal protein S21	0.62
sau:SA0133	*dra*	Deoxyribose-phosphate aldolase	0.59
sau:SA0162	*aldA*	Putative aldehyde dehydrogenase aldA	0.58
sau:SA0957		UPF0637 protein SA0957	0.56
sau:SA1053	*rpoZ*	DNA-directed RNA polymerase subunit omega	0.51
sau:SA0204	*acpD*	FMN-dependent NADH-azoreductase	0.49
sau:SA0232	*lctE*	L-lactate dehydrogenase 1	0.47
sau:SA0307	*nanE*	Putative N-acetylmannosamine-6-phosphate 2-epimerase	0.43
sau:SA1305	*hu*	DNA-binding protein HU	0.42
sau:SA0365	*ahpF*	Alkyl hydroperoxide reductase subunit F	0.42
sau:SA1922	*rpmE2*	50S ribosomal protein L31 type B	0.41
sau:SA0366	*ahpC*	Alkyl hydroperoxide reductase subunit C	0.4
sau:SA0367	*nfrA*	NADPH-dependent oxidoreductase	0.4
sau:SA1081	*rpsP*	30S ribosomal protein S16	0.39
sau:SA1471	*rpmA*	50S ribosomal protein L27	0.39
sau:SA1116	*rpsO*	30S ribosomal protein S15	0.38
sau:SA2036	*rplX*	50S ribosomal protein L24	0.34
sau:SA0468	*hprT*	Hypoxanthine-guanine phosphoribosyltransferase	0.34
sau:SA0478	*pdxT*	Glutamine amidotransferase subunit pdxT	0.32
sau:SA0488	*syc*	Cysteinyl-tRNA synthetase	0.32
sau:SA0573	*sarA*	Transcriptional regulator sarA	0.26
sau:SA2029	*rplO*	50S ribosomal protein L15	0.25
sau:SA2423	*clfB*	Clumping factor B	0.25
sau:SA1901	*fabZ*	(3R)-hydroxymyristoyl-[acyl-carrier-protein] dehydratase	0.24
sau:SA0512	*ilvE*	Probable branched-chain-amino-acid aminotransferase	0.24
sau:SA0520	*sdrD*	Serine-aspartate repeat-containing protein D	0.24
sau:SA0480	*ctsR*	Transcriptional regulator ctsR	0.22
sau:SA1172	*guaC*	GMP reductase	0.22
sau:SA0537	*thiD*	Phosphomethylpyrimidine kinase	0.22
sau:SA0544		UPF0447 protein MW0542; heme peroxidase	0.22
sau:SA1583	*rot*	HTH-type transcriptional regulator rot	0.2
sau:SA0772	*Y772*	UPF0337 protein SA0772	0.2
sau:SA0818	*rocD*	Ornithine aminotransferase 2	0.2
sau:SA0977	*isdA*	Iron-regulated surface determinant protein A	0.2
sau:SA0942	*def*	Peptide deformylase	0.19
sau:SA1032	*sepF*	Cell division protein sepF	0.18
sau:SA1468	*ruvA*	Holliday junction ATP-dependent DNA helicase ruvA	0.17
sau:SA2046	*rplD*	50S ribosomal protein L4	0.17
**Log Phase**			
**ID**	**Gene**	**Annotation**	**emPAI**
sau:SA0944	*phdB*	Pyruvate dehydrogenase E1 component subunit beta	2.39
sau:SA1414	*rpsT*	30S ribosomal protein S20	2.06
sau:SA2033	*rplF*	50S ribosomal protein L6	1.91
sau:SA0723	*clpP*	ATP-dependent Clp protease proteolytic subunit	1.67
sau:SA0504	*rpsG*	30S ribosomal protein S7	1.2
sau:SA1382	*sodA*	Superoxide dismutase [Mn/Fe] 1	1.18
sau:SA0729	*tpi*	Triosephosphate isomerase	1.18
sau:SA1663		UPF0342 protein SA1663	1.17
sau:SA0366	*ahpC*	Alkyl hydroperoxide reductase subunit C	0.95
sau:SA0456	*spoVG*	Putative septation protein spoVG	0.83
sau:SA2036	*rplX*	50S ribosomal protein L24	0.81
sau:SA1073	*fabD*	Malonyl CoA-acyl carrier protein transacylase	0.7
sau:SA1930	*rpoE*	Probable DNA-directed RNA polymerase subunit delta	0.66
sau:SA1113	*rbfA*	Ribosome-binding factor A	0.66
sau:SA2312	*ddh*	D-lactate dehydrogenase	0.63
sau:SA1404	*rpsU*	30S ribosomal protein S21	0.62
sau:SA0856	*spxA*	Regulatory protein spx	0.56
sau:SA2029	*rplO*	50S ribosomal protein L15	0.56
sau:SA1901	*fabZ*	(3R)-hydroxymyristoyl-[acyl-carrier-protein] dehydratase	0.53
sau:SA0719	*trxB*	Thioredoxin reductase	0.53
sau:SA2039	*rpmC*	50S ribosomal protein L29	0.51
sau:SA2026	*infA*	Translation initiation factor IF-1	0.49
sau:SA0245	*ispD*	2-C-methyl-D-erythritol 4-phosphate cytidylyltransferase 2	0.49
sau:SA0918	*purC*	Phosphoribosylaminoimidazole-succinocarboxamide synthase	0.49
sau:SA0941		UPF0356 protein SA0941	0.46
sau:SA0354	*rpsR*	30S ribosomal protein S18	0.43
sau:SA1653	*traP*	Signal transduction protein TRAP	0.43
sau:SA1305	*hu*	DNA-binding protein HU	0.42
sau:SA1359	*EF-P*	Elongation factor P	0.41
sau:SA0942	*pdf1*	Peptide deformylase	0.41
sau:SAS074		UPF0457 protein SA1975	0.4
sau:SA1081	*rpsP*	30S ribosomal protein S16	0.39
sau:SA2043	*rpsS*	30S ribosomal protein S19	0.38
sau:SA2399		Fructose-bisphosphate aldolase class 1	0.38
sau:SA0707		Uncharacterized protein SAB0704	0.37
sau:SA0128	*sodM*	Superoxide dismutase [Mn/Fe] 2	0.36
sau:SA1717	*gatC*	tRNA(Asn/Gln) amidotransferase subunit C	0.36
sau:SA0352	*rpsF*	30S ribosomal protein S6	0.34
sau:SA0855	*trpS*	Tryptophanyl-tRNA synthetase	0.34
sau:SA0437		UPF0133 protein SAB0428	0.34
sau:SA2427	*arcB*	Ornithine carbamoyltransferase catabolic	0.33
sau:SA2127	*rpi*	Ribose-5-phosphate isomerase A	0.32
sau:SA1074	*fabG*	3-oxoacyl-[acyl-carrier-protein] reductase	0.31
sau:SA0160		Heme-degrading monooxygenase isdI	0.31
sau:SA2037	*rplN*	50S ribosomal protein L14	0.3
sau:SA2089	*sarR*	HTH-type transcriptional regulator sarR	0.29
sau:SA2022	*rplQ*	50S ribosomal protein L17	0.29
sau:SA0473	*folB*	Dihydroneopterin aldolase	0.29
sau:SA0108	*sarH1*	HTH-type transcriptional regulator sarS	0.27
sau:SA0573	*sarA*	Transcriptional regulator sarA	0.26
**Stationary Phase**			
**ID**	**Gene**	**Annotation**	**emPAI**
sau:SA0992	*trxA*	Thioredoxin	4.91
sau:SA0295		30 kDa neutral phosphatase (Fragment)	3.92
sau:SA0873		UPF0477 protein SA0873	2.57
sau:SA1178		UPF0154 protein SSP1415	2.14
sau:SA1305	*hu*	DNA-binding protein HU	1.85
sau:SA1663		UPF0342 protein SA1663	1.82
sau:SA2043	*rpsS*	30S ribosomal protein S19	1.6
sau:SA1067	*rpmB*	50S ribosomal protein L28	1.55
sau:SA0456	*spoVG*	Putative septation protein spoVG	1.47
sau:SA1909	*atpF*	ATP synthase subunit b	1.46
sau:SA1709	*ftn*	Ferritin	1.45
sau:SA0160	*isdI*	Heme-degrading monooxygenase isdI	1.24
sau:SA2062	*sarV*	HTH-type transcriptional regulator sarV	1.1
sau:SA0760		Glycine cleavage system H protein	1.09
sau:SA0108	*sarH1*	HTH-type transcriptional regulator sarH1	1.04
sau:SAS078	*rpmJ*	50S ribosomal protein L36	1.01
sau:SA1904	*atpC*	ATP synthase epsilon chain	1.01
sau:SA0032	*bleO*	Bleomycin resistance protein	0.99
sau:SA0494	*nusG*	Transcription antitermination protein nusG	0.98
sau:SA0478	*pdxT*	Glutamine amidotransferase subunit pdxT	0.97
sau:SA2038	*rpsQ*	30S ribosomal protein S17	0.94
sau:SA0245	*ispD*	2-C-methyl-D-erythritol 4-phosphate cytidylyltransferase 2	0.94
sau:SA1901	*fabZ*	(3R)-hydroxymyristoyl-[acyl-carrier-protein] dehydratase	0.9
sau:SA1256	*msrB*	Peptide methionine sulfoxide reductase msrB	0.89
sau:SA0128	*sodM*	Superoxide dismutase [Mn/Fe] 2	0.85
sau:SA1019		Uncharacterized N-acetyltransferase SA1019	0.85
sau:SA0437		UPF0133 protein SAB0428	0.81
sau:SA1074	*fabG*	3-oxoacyl-[acyl-carrier-protein] reductase	0.72
sau:SA2431	*isaB*	Immunodominant staphylococcal antigen B	0.72
sau:SA1041	*pyrR*	Bifunctional protein pyrR	0.7
sau:SA2089	*sarR*	HTH-type transcriptional regulator sarR	0.65
sau:SAP018	*arsC*	Protein arsC	0.59
sau:SA2040	*rplP*	50S ribosomal protein L16	0.54
sau:SA2266		Uncharacterized oxidoreductase SAR2567	0.54
sau:SA1529		UPF0173 metal-dependent hydrolase SA1529	0.52
sau:SA1146	*bsaA*	Glutathione peroxidase homolog bsaA	0.47
sau:SA1076	*rnc*	Ribonuclease 3	0.46
sau:SA0774		Probable ABC transporter ATP-binding protein	0.46
sau:SA0941		UPF0356 protein SA0941	0.46
sau:SA1461	*apt*	Adenine phosphoribosyltransferase	0.44
sau:SA2392	*panB*	3-methyl-2-oxobutanoate hydroxymethyltransferase	0.44
sau:SA1206	*femA*	Aminoacyltransferase femA	0.44
sau:SA0354	*rpsR*	30S ribosomal protein S18	0.43
sau:SA0934	*ptsH*	Phosphocarrier protein HPr	0.42
sau:SA1032	*sepF*	Cell division protein sepF	0.4
sau:SA0470	*hsp33*	33 kDa chaperonin	0.4
sau:SA1471	*rpmA*	50S ribosomal protein L27	0.39
sau:SA1081	*rpsP*	30S ribosomal protein S16	0.39
sau:SA0704		UPF0230 protein	0.39
sau:SA0826	*spsB*	Signal peptidase IB	0.39

blue: ribosomal protein; red: Hu; yellow: oxidoreductases; green: global regulator; grey: fatty acid metabolism.

**Table 2:** Genes with higher expression in WT(+PQ) than ∆*mrgA*(+PQ). Oxidative stress was given by 20 µM PQ at 37 °C for 30 min to log phase cells. Transcriptome was analyzed by a standard procedure by using GeneChip (Affymetrix).

Log-Difference WT(+PQ) /DmrgA(+PQ)	Gene Name	N315 SA Number	Annotation/Similarity
4.04	*mrgA(dps)*	*SA1941*	MrgA, Dps family protein
2.07	*lrgB*	*SA0253*	antiholin-like protein LrgB
1.86	*/*	*SA2133*	hypothetical protein
1.65	*oppB*	*SA0853*	oligopeptide ABC transporter permease
1.59	*arcB*	*SA2427*	ornithine carbamoyltransferase
1.54	*sen*	*SA1643*	enterotoxin SeN (in pathogenicity island, SaPIn3)
1.53	*/*	*SA2470*	histidinol dehydrogenase
1.51	*/*	*SA2417*	nisin susceptibility-associated two-component sensor histidine kinase
1.50	*/*	*SA2264*	hypothetical protein
1.49	*hisZ*	*SA2472*	ATP phosphoribosyltransferase regulatory subunit/ His-tRNA synthase
1.48	*/*	*SA2454*	acetyltransferase
1.35	*/*	*SA2429*	ArgR family transcriptional regulator
1.31	*/*	*SA0667*	7-cyano-7-deazaguanine synthase
1.30	*pyrF*	*SA1047*	orotidine 5′-phosphate decarboxylase
1.30	*/*	*SA0846*	oligopeptide transport system permease OppC
1.29	*/*	*SA1760*	holin-like protein (in phage phiN315)
1.28	*/*	*SA1807*	mobile element associated protiein (in phage phiN315)
1.26	*/*	*SA0804*	Na+/H+ antiporter family protein
1.22	*lrgA*	*SA0252*	murein hydrolase regulator LrgA
1.22	*yent1*	*SA1645*	enterotoxin Yent1 (in pathogenicity island, SaPIn3)
1.21	*/*	*SA2469*	histidinol-phosphate aminotransferase
1.20	*/*	*SA0582*	monovalent cation/H+ antiporter subunit E
1.15	*purC*	*SA0918*	phosphoribosylaminoimidazole-succinocarboxamide synthase
1.11	*/*	*SA2189*	Ferrochelatase family / cobalamin biosynthesis CbiX/ transcriptional regulator NirR
1.09	*ureE*	*SA2085*	urease accessory protein UreE
1.09	*/*	*SA1768*	phage tail protein (in phage phiN315)
1.03	*/*	*SA1636*	hypothetical protein
1.02	*/*	*SA1675*	amino acid ABC transporter permease/substrate-binding protein
1.02	*nrdD*	*SA2410*	anaerobic ribonucleoside triphosphate reductase
1.02	*ssp*	*SA0744*	secretory extracellular matrix and plasma binding protein
1.01	*/*	*SA0324*	mepB family protein
1.00	*clfB*	*SA2423*	clumping factor B

blue: holin, anti-holin; red: virulence; yellow: transcription regulator; green: nucleic acid metabolism.

**Table 3:** Genes with lower expression in WT(+PQ) than ∆*mrgA*(+PQ). Oxidative stress was given by 20 µM PQ at 37 °C for 30 min to log phase cells. Transcriptome was analyzed by a standard procedure by using GeneChip (Affymetrix).

Log-Difference WT(+PQ) /DmrgA(+PQ)	Gene Name	N315 SA Number	Annotation/Similarity
−3.03	*spa*	*SA0107*	immunoglobulin G binding protein A
−2.41	*/*	*SA0080*	membrane protein similar to sulfite exporter TauE/SafE family protein
−2.35	*/*	*SA0100*	Na/Pi cotransporter family protein
−2.31	*sirC*	*SA0109*	iron compound ABC transporter permease SirC
−1.92	*/*	*SA0090*	hypothetical protein
−1.90	*sarH1*	*SA0108*	staphylococcal accessory regulator H1
−1.87	*lacC*	*SA1995*	tagatose-6-phosphate kinase
−1.84	*/*	*SA0085*	hypothetical protein
−1.81	*/*	*SA0061*	(in Staphylococcus Cassette Chromosome, SCC)
−1.81	*/*	*SA0077*	serine/threonine protein kinase (in Staphylococcus Cassette Chromosome, SCC)
−1.78	*lctP*	*SA0106*	L-lactate permease
−1.73	*/*	*SA2092*	AraC family transcriptional regulator
−1.68	*/*	*SA0102*	myosin-cross-reactive MHC class-II like protein
−1.67	*lpl8*	*SA0404*	lipoprotein encoded in pathogenicity island (in pathogenicity island, SaPIn2)
−1.63	*/*	*SA0124*	capsular polysaccharide biosynthesis glycosyltransferase TuaA
−1.61	*/*	*SA0120*	SbnI, siderophore biosynthesis protein
−1.54	*/*	*SA2230*	fmtA-like protein/ beta lactamase
−1.50	*/*	*SA0085*	tRNA-dihydrouridine synthase
−1.45	*/*	*SA0099*	transmembrane efflux pump protein
−1.43	*vraA*	*SA0533*	long chain fatty acid-CoA ligase vraA
−1.42	*/*	*SA2303*	ABC transporter permease protein
−1.42	*/*	*SA0097*	AraC/XylS family transcriptional regulator
−1.38	*sarY*	*SA2091*	staphylococcal accessory regulator Y
−1.21	*/*	*SA0105*	hypothetical protein
−1.20	*/*	*SA1826*	pathogenicity island protein (in pathogenicity island, SaPIn1)
−1.18	*/*	*SA2274*	hypothetical protein
−1.17	*/*	*SA2302*	ABC transporter ATP-binding protein
−1.12	*/*	*SA0087*	tfoX N-terminal domain protein
−1.08	*/*	*SA0078*	hypothetical protein
−1.07	*/*	*SA0037*	MaoC domain-containing protein (in Staphylococcus Cassette Chromosome, SCC)
−1.06	*/*	*SA0536*	hypothetical protein
−1.05	*/*	*SA2154*	hypothetical protein
−1.05	*/*	*SA0088*	hypothetical protein
−1.05	*lacA*	*SA1997*	galactose-6-phosphate isomerase subunit LacA
−1.04	*sarV*	*SA2062*	staphylococcal accessory regulator V
−1.04	*hisG*	*SA2471*	ATP phosphoribosyltransferase catalytic subunit
−1.03	*/*	*SAS028*	hypothetical protein
−1.00	*sodM*	*SA0128*	superoxide dismutase
−1.00	*fmhA*	*SA2199*	fmhA protein (FemAB like protein,)

red: virulence; yellow: transcription regulator; pale red: iron metabolism; pale blue: oxidative stress related.
